# Therapeutics of Iodol

**Published:** 1889-04

**Authors:** 


### Therapeutics of Iodol.

Prof. Dante Cervesato, of Padua, has communicated to *Lo Sperimentale*, September, 1888, his experiences with the therapeutic use of iodol in internal diseases. It was first tried in scrofulosis, in all forms of which it acted well. It was most active in the torpid form, especially in torpid, swollen lymph glands which had not yet suppurated—and not only in the peripheral glands but also in the bronchial and mesenteric. It was less favorable in its action upon scrofulosis of the mucous membranes, especially of the nose and ear. It has very little influence upon scrofulous dermatoses. It was employed internally in doses of seven and a half, fifteen, and twenty-two and a half grains in older children, and the treatment could be kept up without harm for two or three months uninterruptedly. Iodoform ointment (one part to fifteen of vaseline) and insufflations of iodoform were useful adjuncts. The iodol was better borne; not only did no digestive disturbances occur, but the digestion even improved. In diseases of the respiratory organs iodol was given in doses of fifteen to forty-five grains a day, in addition to inhalations with the following solution : One part of iodol is dissolved in five parts of warm absolute alcohol ; to the filtered solution ten parts of glycerine at (60°—70°) are added. Before the solution cools ten parts of water are added with constant stirring. The iodol remains proportionately suspended for a long time. Four drachms of the emulsion are used for each inhalation, which is given two or three times a day. He has observed marked improvement in primary tuberculosis of the larynx. In acute and chronic catarrhal laryngitis, and in different forms of bronchitis, the results were brilliant. In tertiary syphilis it was employed with very favorable results. Two patients with extensive ulcers on the palate and pharynx recovered after the internal and local use of iodol for two months. Internally iodol was given in doses of fifteen to forty-five grains ; the following solution was used locally :

| | |
|---|---|
| Iodol - - - - - - | gr. xv. |
| Alcohol - - - - - - | f ʒ iv. |
| Glycerine - - - - - | f ʒ viii. |

M.

In a case of tertiary syphilis with lesions of the liver and of the larynx, the internal use of iodol achieved wonders. No iodisin nor any other disagreeable accompanying symptoms whatsoever occurred in any of the cases.—*Wiener med. Presse*, December 2, 1888.

